# Use of a Pre-Trained Neural Network for Automatic Classification of Arterial Doppler Flow Waveforms: A Proof of Concept

**DOI:** 10.3390/jcm10194479

**Published:** 2021-09-28

**Authors:** Antoine Guilcher, Damien Laneelle, Guillaume Mahé

**Affiliations:** 1 Vascular Medicine Unit, CHU Rennes, 35000 Rennes, France; antoine.guilcher@chu-rennes.fr; 2 Vascular Medicine Unit, CHU Caen-Normandie, 14033 Caen, France; laneelle-d@chu-caen.fr; 3 Clinical Investigation Center, University Rennes, INSERM CIC 1414, 35033 Rennes, France; 4 M2S-EA 7470, University Rennes, 35170 Bruz, France

**Keywords:** Doppler waveform, peripheral artery disease, neural network classification

## Abstract

Background: Arterial Doppler flow waveform analysis is a tool recommended for the management of lower extremity peripheral arterial disease (PAD). To standardize the waveform analysis, classifications have been proposed. Neural networks have shown a great ability to categorize data. The aim of the present study was to use an existing neural network to evaluate the potential for categorization of arterial Doppler flow waveforms according to a commonly used classification. Methods: The Pareto efficient ResNet-101 (ResNet-101) neural network was chosen to categorize 424 images of arterial Doppler flow waveforms according to the Simplified Saint-Bonnet classification. As a reference, the inter-operator variability between two trained vascular medicine physicians was also assessed. Accuracy was expressed in percentage, and agreement was assessed using Cohen’s Kappa coefficient. Results: After retraining, ResNet-101 was able to categorize waveforms with 83.7 ± 4.6% accuracy resulting in a kappa coefficient of 0.79 (0.75–0.83) (CI 95%), compared with a kappa coefficient of 0.83 (0.79–0.87) (CI 95%) between the two physicians. Conclusion: This study suggests that the use of transfer learning on a pre-trained neural network is feasible for the automatic classification of images of arterial Doppler flow waveforms.

## 1. Introduction

With an aging population worldwide, lower extremity peripheral artery disease (PAD) has become a major health issue [1,2] with significant financial implications [3,4,5,6]. Consequently, optimization of the management of patients suffering from PAD is a matter of upmost importance.

Along with ankle brachial pressure index (ABI) and toe brachial pressure index (TBI), arterial Doppler waveform analysis is a tool recommended to diagnose and manage PAD [7,8,9]. For years, researchers have described the relationship between waveform contours and PAD severity [10,11,12,13]. Arterial Doppler waveforms are usually described according to the number of phases (i.e., alternance between forward and backward flow) and “sharpness” (i.e., acceleration/deceleration of blood flow) [14,15,16,17]. It is commonly accepted that the greater the number of phases and the sharper the signal the healthier the artery, and vice versa [17,18]. However, the interpretation and description of Doppler waveforms is highly subjective [19].

As a result, an abundance of terms has surfaced in the literature to describe Doppler waveforms, often leading to confusion and misunderstanding between medical staff, resulting in a differing management of PAD [19,20,21]. In order to standardize arterial Doppler waveform description, and improve Doppler waveform analysis, several classifications have been proposed [14,15,16] to categorize arterial flow contours and assess PAD severity. However, currently, none of the classifications propose an exhaustive description of the various arterial Doppler flow waveforms observed by vascular physicians in a clinical setting. Thus, reproducibility errors remain. One method to prevent reproducibility errors is to automate the categorization process.

Since the 1960s there has been a growing emphasis on computer aided diagnosis (CADg) in medicine. An increasingly utilized CADg technique is the application of neural networks in the classification of patients’ conditions [22], including in patients suffering from PAD [23,24]. A neural network trained by experts to categorize arterial Doppler flow waveforms could potentially remove the remaining subjectivity in the classification process. However, creating and training a dedicated neural network is a complex process which requires a specific skill set and computational power, which for many medical teams could be unattainable.

One way to overcome these issues is a technique called transfer learning. Transfer learning utilizes a pre-trained network which is “retrained” to perform a new task. As the architecture of the neural network is already built, only a few parameters have to be adapted, making the technique relatively accessible to a novice user. Among the pre-trained networks freely available are the networks participating in the ImageNet Large-Scale Visual Recognition Challenge (ILSVRC) [25]. These networks are trained on more than a million images from the ImageNet database (http://www.image-net.org, accessed on 18 March 2021) to classify images according to a thousand categories.

The aim of the present study was to use transfer learning on a pre-trained neural network in order to automatically categorize images of arterial Doppler flow waveforms, and to assess its feasibility.

## 2. Materials and Methods

Arterial Doppler waveforms were acquired from patients who attended the vascular clinic of the University Hospital in Rennes from January 2019 to February 2020. The acquisition was performed with Philips iU22, Philips Epiq 7 and Mindray Resona 7 ultrasound machines using a linear probe in pulse-wave Doppler mode. Waveforms were selected only if they displayed at least one full cardiac cycle, if they did not exhibit signal loss or high velocity shift artifact, if the pulse-wave Doppler spectrum was not suffering from an excess of overgain and if they were not masked by low frequency noise. For each of the selected waveforms, a full cardiac cycle was then manually selected using a custom-made software developed using MATLAB (version R2190b, The MathWorks, Natick, MA USA). The complete dataset consisted of 424 waveforms, each representing a single cardiac cycle. These waveforms fitted 9 categories after categorization by a vascular physician according to the simplified Saint-Bonnet classification [14]. One hundred and forty-five waveforms were categorized as N, 15 as N-CF, 125 as A, 0 as A-CF, 19 as B, 29 as B-CF, 29 as CD, 35 as CD-CF, 13 as E and 14 as E-CF, according to the simplified Saint-Bonnet classification (Table 1).

### 2.1. Reference Classification

**Figure 1:**
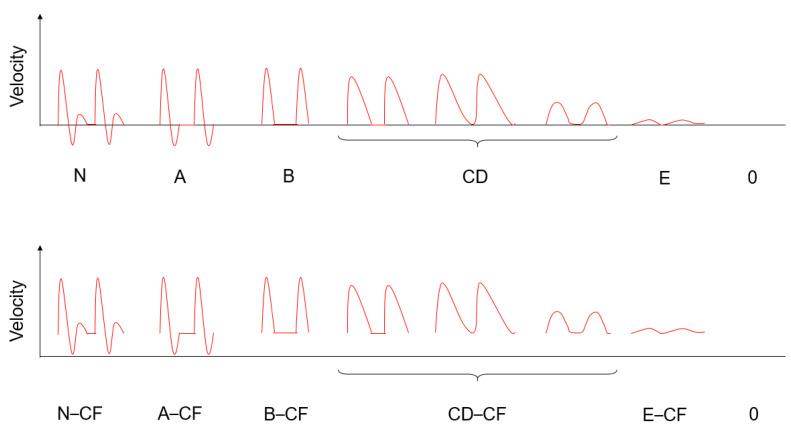
The simplified Saint-Bonnet classification. There was no waveform in categories A–CF and 0 in the present study.

The simplified Saint-Bonnet classification (Figure 1) was chosen as the reference classification for two reasons: (i) The College of the French Vascular Medicine Teachers (Collège des Enseignants de Médecine Vasculaire, CEMV) recommends the use of the simplified Saint-Bonnet Classification in its most recent guidelines [9,26] and (ii) The simplified Saint-Bonnet Classification categories describe extensive types of arterial Doppler flow waveform representing different progressing grades of PAD severity [14].

### 2.2. Network Choice and Settings

The neural network was chosen among Pareto efficient networks (i.e., the best of its class for the combination of accuracy and computational time) participating in the ILSVRC. The computational power available for the study being quite modest (AMD 3700X CPU and NVIDIA GTX 1660 Super GPU with 6GB of RAM), the ResNet-101 network was selected. Training time for the task, using transfer learning, was around three and a half minutes.

Neural network training, testing and data processing were completed using MATLAB software and its Deep Learning Toolbox (version R2190b, The MathWorks, Natick, MA, USA).

Neural networks consist of several layers. An input layer, which matches the data format, stem layers which condition the data, hidden layers which extract the key characteristics of the data and final layers which perform the classification. As ResNet-101 accepts only 224 × 224 pixel images as inputs, the grayscale images of the selected waveforms were resized to match this format. Doppler flow waveform images were padded with black colored pixels (either along the x-axis or the y-axis) in order to obtain a square image, thus avoiding additional distortion. These images where then scaled to 224 × 224 pixels. In addition, as ResNet-101 is designed to sort images into a thousand object categories, much greater than what is required for the objective of this study, two of the final layers of the neural network were replaced by new classification layers. The new layers matched the number of arterial Doppler flow waveform categories found within the 424 selected waveforms according to the simplified Saint-Bonnet Classification (9 categories in the present study).

Default values were used for all parameters for transfer learning with the exception of: (i) the number of epochs (a full pass of the data though the network), which was set to 7 at which point the network accuracy was plateauing; (ii) the mini batch size (a sub sample of the dataset used to estimate and reduce prediction errors) which was set to 8, meeting Masters and Luschi criteria [27], with samples being shuffled before each epoch to avoid discarding the same data every epoch as the number of waveforms in the training set was not necessarily a multiple of 8; and (iii) the initial learning rate which was set to 3 × 10^−4^.

Several measures were implemented to reduce overfitting. Due to the small size of the dataset, no training was performed on the stem layers of Resnet101. The number of maximum epochs was chosen so that very little training would be done after the network accuracy had started to converge. Finally, waveforms of the training set were randomly modified by applying small offset and scaling coefficients, a process known as data augmentation [28].

### 2.3. Network Training and Validation

**Figure 2:**
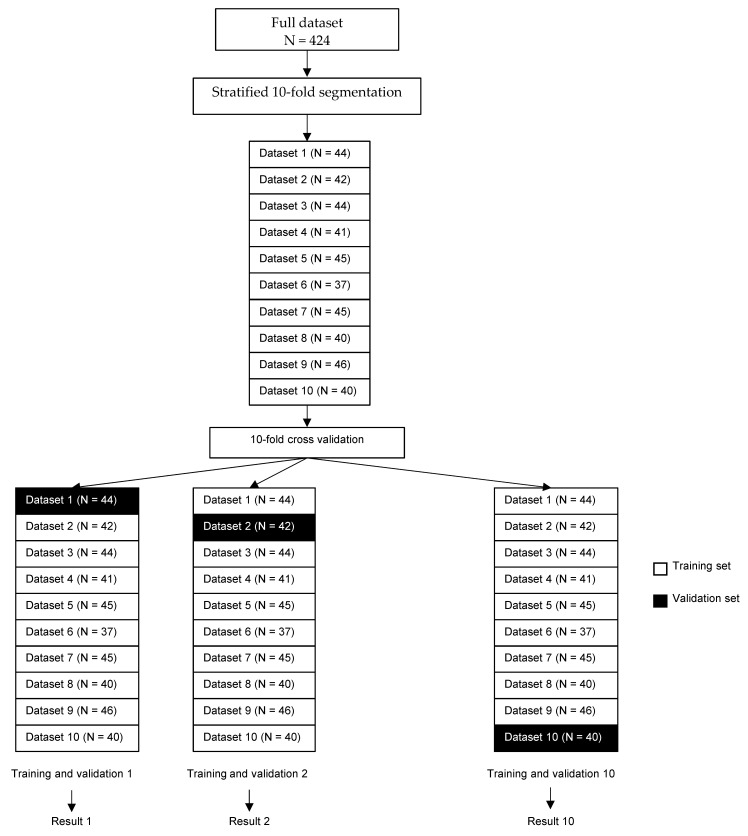
Illustration of the 10-fold cross-validation process. Legend: For the 10-fold cross validation, 10 sub datasets were used and for each of the 10 training and validation procedures, training was performed on 9 sub datasets (white boxes) and tested on the remaining sub dataset (black box). The subset used for testing changed between each procedure. Average accuracy and agreement compared with a vascular physician (physician 1) were calculated.

The small size of the dataset and the uneven sample size across categories lead to a risk of unstable prediction when training neural networks. For this reason, and the fact that it provides a better estimate of how the neural network will perform in a real-life setting, k-fold cross validation (with *k* = 10) was used to assess the accuracy of the neural network in categorizing arterial Doppler flow waveforms [29]. Ten stratified sub-datasets were randomly generated from the original dataset. Training was performed using 9 of the 10 datasets, with validation performed on the remaining, unknown to the network, dataset. The process was repeated 10 times, changing the validation set and the training sets at each iteration (Figure 2).

### 2.4. Comparison with Humans

In order to have a point of reference on which to assess the neural network performance, two vascular physicians (physician 1 and physician 2) familiar with the simplified Saint-Bonnet classification (daily use for at least 2 years) categorized the same 424 waveforms used as inputs for the neural network. The categorization was performed according to the simplified Saint-Bonnet classification, and the agreement between the two physicians was assessed.

### 2.5. Statistics

The accuracy of the neural network classification was expressed in percentage ± standard deviation (SD). The agreement between the neural network and the reference vascular physician, as well as the agreement between the two trained vascular medicine physicians, were assessed with Cohen’s Kappa coefficient [30] using MedCalc (version 18.5, MedCalc Software, Ostend, Belgium).

## 3. Results

In comparison with the waveform categorization performed by physician 1, the average accuracy of the ResNet-101 neural network to categorize arterial Doppler flow waveforms, after the transfer learning process, was 83.7 ± 4.6%. The resulting Cohen’s Kappa coefficient was 0.79 (0.75–0.83) (CI 95%), indicating a moderate agreement [30]. Specifically, 86.9% of the N waveforms, 40.0% of the N-CF waveforms, 94.4% of the A waveforms, 68.4% of the B waveforms, 69.0% of the B-CF waveforms, 72.4% of the CD waveforms, 80.0% of the CD-CF waveforms, 76.9% of the E waveforms and 92.9% of the E-CF waveforms were categorized correctly (Table 2).

**Figure 3:**
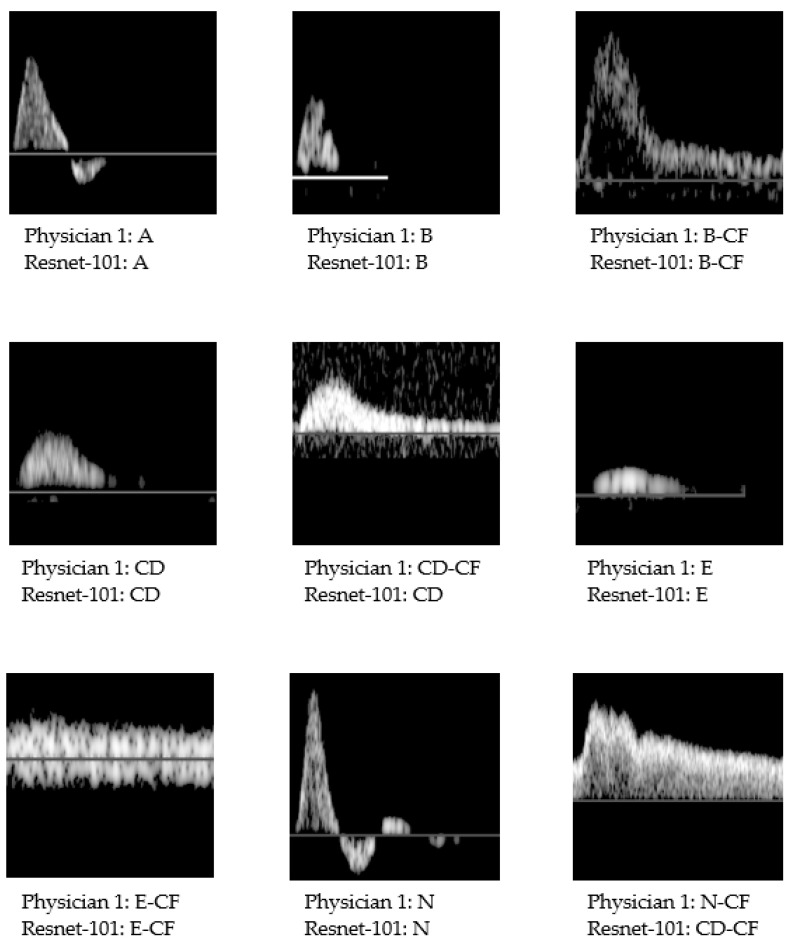
Example of waveforms categorized by ResNet-101 in comparison with the waveform categorization performed by physician 1, according to the simplified Saint-Bonnet classification. Physician 1: reference physician.

An example of the neural network waveform categorization can be seen in Figure 3.

When comparing the categorization of the waveforms performed by the two trained vascular physicians, 86.5% (*n* = 367) of the waveforms were categorized identically. The resulting Cohen’s Kappa coefficient between the two physicians was 0.83 (0.79–0.87) (CI 95%), indicating a strong agreement [30]. Specifically, 81.4% of the N waveforms, 93.3% of the N-CF waveforms, 98.4% of the A waveforms, 78.9% of the B waveforms, 51.7% of the B-CF waveforms, 72.4% of the CD waveforms, 97.1% of the CD-CF waveforms, 100% of the E waveforms and 100% of the E-CF waveforms were categorized identically (Table 3).

## 4. Discussion

Transfer learning using the ResNet-101 network enabled the categorization of arterial Doppler flow waveforms according to the simplified Saint-Bonnet classification with an accuracy of 83.7 ± 4.6% and a Cohen’s Kappa coefficient of 0.79 (0.75–0.83) compared with a vascular medicine physician familiar with the simplified Saint-Bonnet classification. Although this agreement does not reach that obtained between two trained vascular physicians (Kappa = 0.83 (0.79–0.87), strong agreement) it is very similar and without any potential reproducibility error. These results are in keeping with those of previous studies where neural networks were used to diagnose PAD [23,24] and the prospect of perfect reproducibility is of importance, especially as an arterial Doppler waveform description is frequently debated amongst vascular medicine physicians [17]. Neural networks could indeed provide an objective approach for categorization and a description of arterial Doppler waveforms.

The detailed results of the waveform classification performed by ResNet-101 (Table 2) indicates that the chosen neural network underperformed for the N-CF waveform category with 53% of these waveforms being incorrectly categorized as B-CF or CD-CF. This result could indicate that the neural network may have problems distinguishing between waveforms exhibiting a continuous flow. However, the results for waveform types B-CF (69.0%), CD-CF (80.0%) and E-CF (92.9%) demonstrate greater accuracy, with only one waveform wrongly categorized as N-CF. The neural network behaves as if it is overlooking the N-CF category. This is an expected result, as the N-CF category contains a low sample number (15 waveforms). Indeed, to optimize the training process, a neural network requires a large training set to ensure all waveforms observed in clinical practice are represented, and the number of samples within each category must be similar in order to limit bias towards, or against, certain categories. Therefore, the results of the present study could be improved by introducing a larger and more balanced dataset.

The main discrepancy in waveform categorization between the two vascular physicians resulted from difficulties in differentiating between B-CF and CD-CF waveforms. This disagreement is typical in general practice, as these two categories are sequential in the simplified Saint-Bonnet classification (Figure 1) and in terms of degree of severity of PAD. The main difference between B-CF and CD-CF categories resides in a change of the upstroke and the downstroke of the systolic velocity peak. This change is progressive, with no set threshold and is thus prone to inter-operator reproducibility error.

Concerning the simplified Saint-Bonnet classification, the results of the present study suggest that its use may be highly reproductible with a Cohen’s Kappa coefficient of 0.83 (0.79–0.87), indicating a strong inter-operator agreement. The results also highlight the fact that some categories, such as A-CF, may be theoretical or have a very low prevalence in a population of patients consulting vascular clinics.

Although the use of the simplified Saint-Bonnet classification is recommended by The College of the French Vascular Medicine Teachers, it has not been endorsed internationally. However, compared to other classifications [15,16] the simplified Saint-Bonnet classification includes the most categories. Therefore, the use of a neural network to categorize arterial Doppler waveforms according to other classifications, with an equal or a lower number of categories, should provide similar accuracy, as long as those categories are of relevance.

Moving forward, the ideal situation would be to generate a large, freely accessible database of waveforms. This would enable a better description of waveform categories, potentially leading to the creation of a more robust classification, which could be utilized globally. It would also provide larger training and testing datasets for more precise automatic categorization. To achieve this aim, vascular medicine societies across the world should be encouraged to collaborate and share waveform data.

The present study has several limitations. As previously discussed, the dataset used was suboptimal due to the small number of waveforms and the lack of equality between waveform categories. Furthermore, as this study was designed as a proof of concept, the patient characteristics were unknown and although, in theory, a large number of different waveforms should correlate to differing underlying conditions and degree of disease, a selection bias cannot be excluded. Finally, as mentioned previously, the neural network was only trained to categorize waveforms according to the simplified Saint Bonnet classification, and therefore the results from this study cannot be extrapolated to other classifications.

## 5. Conclusions

Computer-aided diagnostics will undoubtedly be a part of the future of medicine, and medical practitioners should not only be “end users”, but must also play a part in the development of such tools for clinical practice. The results of the present study suggest that it is possible to use an existing neural network with the transfer learning technique to categorize arterial Doppler waveform with good accuracy. The creation of a worldwide, freely accessible, arterial Doppler waveform database would greatly improve these preliminary results. Such a tool could then be key in standardizing arterial Doppler waveforms analysis and thus improve PAD management.

## Figures and Tables

**Table 1:** Full dataset waveform distribution according the simplified Saint-Bonnet classification.

Category	Number	%
N	145	34.2
N-CF	15	3.5
A	125	29.5
A-CF	0	0.0
B	19	4.5
B-CF	29	6.8
CD	29	6.8
CD-CF	35	8.3
E	13	3.1
E-CF	14	3.3

**Table 2:** Comparison of ResNet-101 waveform categorization with the reference vascular medicine physician according to the simplified Saint-Bonnet classification.

	Physician 1								
ResNet-101	N	N-CF	A	B	B-CF	CD	CD-CF	E	E-CF
**N**	**126**	1	4	0	0	0	0	0	0
**N-CF**	7	**6**	0	0	0	0	1	0	0
**A**	7	0	**118**	2	1	1	0	0	0
**B**	0	0	1	**13**	0	4	0	0	0
**B-CF**	2	3	0	1	**20**	0	4	0	0
**CD**	3	0	2	3	0	**21**	2	2	0
**CD-CF**	0	5	0	0	7	1	**28**	0	1
**E**	0	0	0	0	1	2	0	**10**	0
**E-CF**	0	0	0	0	0	0	0	1	**13**
**Agreement (%)**	**86.9**	**40.0**	**94.4**	**68.4**	**69.0**	**72.4**	**80.0**	**76.9**	**92.9**

ResNet-101 waveform categorization pooled from the 10-fold cross validation tests; Physician 1: reference physician. Numbers in bold represent the number of arterial Doppler waveforms that were in the same category according to the Physician 1 and the ResNet-101.

**Table 3:** Comparison of waveform categorization between the two trained vascular medicine physicians according to the simplified Saint-Bonnet classification.

	Physician 1								
Physician 2	N	N-CF	A	B	B-CF	CD	CD-CF	E	E-CF
**N**	**118**	0	2	0	0	0	0	0	0
**N-CF**	27	**14**	0	0	0	0	0	0	0
**A**	0	0	**123**	0	0	0	0	0	0
**B**	0	0	0	**15**	0	6	0	0	0
**B-CF**	0	1	0	0	**15**	0	1	0	0
**CD**	0	0	0	4	0	**21**	0	0	0
**CD-CF**	0	0	0	0	14	1	**34**	0	0
**E**	0	0	0	0	0	1	0	**13**	0
**E-CF**	0	0	0	0	0	0	0	0	**14**
**Agreement (%)**	**81.4**	**93.3**	**98.4**	**78.9**	**51.7**	**72.4**	**97.1**	**100.0**	**100.0**

Physician 1: reference physician; Physician 2: trained vascular medicine physician. Numbers in bold represent the number of arterial Doppler waveforms that were in the same category according to the Physician 1 and the Physician 2.

## Data Availability

The data that support the findings of this study are available from the corresponding author upon reasonable request.
